# Thymoquinone regulates gene expression levels in the estrogen metabolic and interferon pathways in MCF7 breast cancer cells

**DOI:** 10.3892/ijmm.2013.1563

**Published:** 2013-11-21

**Authors:** MARJANEH MOTAGHED, FAISAL MUTI AL-HASSAN, SHAHRUL SAHUL HAMID

**Affiliations:** Oncology and Radiological Science Cluster, Advanced Medical and Dental Institute, Universiti Sains Malaysia, Kepala Batas, Penang 13200, Malaysia

**Keywords:** thymoquinone, cDNA microarray, gene expression

## Abstract

New drugs are continuously being developed for the treatment of patients with estrogen receptor-positive breast cancer. Thymoquinone is one of the drugs that exhibits anticancer characteristics based on *in vivo* and *in vitro* models. This study further investigates the effects of thymoquinone on human gene expression using cDNA microarray technology. The quantification of RNA samples was carried out using an Agilent 2100 Bioanalyser to determine the RNA integrity number (RIN). The Agilent Low Input Quick Amplification Labelling kit was used to generate cRNA in two-color microarray analysis. Samples with RIN >9.0 were used in this study. The universal human reference RNA was used as the common reference. The samples were labelled with cyanine-3 (cye-3) CTP dye and the universal human reference was labelled with cyanine-5 (cye-5) CTP dye. cRNA was purified with the RNeasy Plus Mini kit and quantified using a NanoDrop 2000c spectrophotometer. The arrays were scanned data analysed using Feature Extraction and GeneSpring software. Two-step qRT-PCR was selected to determine the relative gene expression using the High Capacity RNA-to-cDNA kit. The results from Gene Ontology (GO) analysis, indicated that 8 GO terms were related to biological processes (84%) and molecular functions (16%). A total of 577 entities showed >2-fold change in expression. Of these entities, 45.2% showed an upregulation and 54.7% showed a downregulation in expression. The interpretation of single experiment analysis (SEA) revealed that the cytochrome P450, family 1, subfamily A, polypeptide 1 (*CYP1A1*) and UDP glucuronosyltransferase 1 family, polypeptide A8 (*UGT1A8*) genes in the estrogen metabolic pathway were downregulated significantly by 43- and 11-fold, respectively. The solute carrier family 7 (anionic amino acid transporter light chain, xc-system), member 11 (*SLC7A11*) gene in the interferon pathway, reported to be involved in the development of chemoresistance, was downregulated by 15-fold. The interferon-induced protein with tetratricopeptide repeats (*IFIT*)*1*, *IFIT2*, *IFIT3*, interferon, α-inducible protein (*IFI)6* (also known as *G1P3*), interferon regulatory factor 9 (*IRF9, ISGF3*), 2′–5′-oligoadenylate synthetase 1, 40/46 kDa (*OAS1*) and signal transducer and activator of transcription 1 (*STAT1)* genes all showed changes in expression following treatment with thymoquinone. The caspase 10, apoptosis-related cysteine peptidase (*CASP10*) gene was activated and the protein tyrosine phosphatase, receptor type, R (*PTPRR)* and myocyte enhancer factor 2C (*MEF2C*) genes were upregulated in the classical MAPK and p38 MAPK pathways. These findings indicate that thymquinone targets specific genes in the estrogen metabolic and interferon pathways.

## Introduction

The World Health Organization International Agency for Research on Cancer reported that approximately 1.29 million women were diagnosed with breast cancer and >400,000 women succumbed to the disease in 2008 (1). Thymoquinone is an anti-neoplasic agent reported to have medicinal potential in the treatment of breast cancer. The antitumour activity of thymoquinone has been reported in cells derived from ovarian, breast and colon cancers (2). The apoptotic activity of thymoquinone has been reported to induce the total Bax/Bcl-2 ratio in MCF7 (3), HCT-116 (4) and HL-60 cancer cells (5). These findings were based on flow cytometry, western blot analysis and multi-colour fluorescence *in situ* hybridization (mFISH). Therefore, further investigation is required to to determine its effects on human genome expression using cDNA microarray technology.

## Materials and methods

### Complete medium preparation

RPMI-1640 (Invitrogen, Gibco, Carlsbad, CA, USA) medium with L-glutamine was used to culture the MCF7 cell line (ATCC^®^ HTB22™). The medium was supplemented with 10% heat-inactivated fetal bovine serum (Invitrogen/Gibco) and 1 unit penicillin/streptomycin (HyClone, Logan, UT, USA).

### Thymoquinone solution preparation

The 1 mM stock solution of thymoquinone (Sigma-Aldrich, Saint-Quentin-Fallavier, France) was prepared with DMSO (Sigma-Aldrich). The solution was filtered with a 0.02 μm syringe filter (Hydrophilic Ministart; Sartorius AG, Goettingen, Germany).

### Cell culture and treatment

The MCF7 cells were seeded at 3–4×10^6^ cells/well in 96-well plates. They were cultured at 0.5% CO_2_ in a humidified incubator at 37°C (Thermo Scientific, Waltham, MA, USA) for 24 h. The control cells were treated with 0.05% dimethyl sulfoxide alone (Sigma-Aldrich). Four biological replicates from each sample were prepared in separate culture flasks. The samples were treated with 50 μM thymoquinone for 24 h. EDTA [0.25% (w/v) + Trypsin/0.53% Mm] solution was used to detach the cells from the flask surface. The cells were then centrifuged at 13,000 rpm for 10 min. The supernatant was removed and the cells were washed with PBS solution prior to centrifugation at 13,000 rpm for 5 min.

### RNA isolation

RNA was isolated from the MCF7 cells using the RNeasy Plus Mini kit (Qiagen, Valencia, CA, USA) according to the manufacturer’s instructions. The quantity of RNA was measured using a spectrophotometer (NanoDrop 2000c; Thermo Scientific). Samples with the RNA concentration (A_260_/A_280_ ≥1.8 ng/μl) and purity (A_230_/A_260_ ≥2.0 ng/μl) were selected. An Agilent 2100 Bioanalyser was used to determine the RNA integrity number (RIN). The degradation level was identified using the RNA 6000 Nano LabChip kit (Agilent Technologies, Santa Clara, CA, USA). The samples with RIN >9.8 were selected for further analysis.

### Two-colour microarray-based gene expression

The Agilent Low Input Quick Amp Labelling kit (USA) was used to generate cRNA with a sample input of 200 ng total RNA in two-color microarray analysis. The RNA Spike-In kit (Agilent Technologies) was used as an external control (positive control). It monitors and calibrates the linearity, sensitivity and accuracy of the microarray workflow. Spike A Mix with cyanine-3 (cye-3) was used to label the samples (thymoqionone-treated and untreated) and Spike B Mix with cyanine-5 (cye-5) was used to label the internal control (Universal Human Reference RNA, Agilent Technologies). T7 RNA polymerase was used to amplify target material.

The array platform used was 8×60K array SurePrint Technology (Slide Human V1 U252800417900-S01; Interscience, Rockland, MA, USA) and gasket slides with SureHyb Technology. The reference design was selected for the study. Four biological replicates from 50 μM thymoquinone-treated MCF7 and four replicates from untreated MCF7 cells were hybridised against Human Universal Reference RNA using a hybridisation kit (Agilent Technologies). The slide chamber was assembled and placed in rotisserie hybridization oven and rotated at 10 rpm in 65°C for 17 h. The array slide was washed and fluorescent imaging system was used to scan the hybridisation signals (DNA Microarray Scanner, Surescan High-Resolution Technology, Agilent Technologies).

The results were extracted with Feature Extraction Project v10.7.3.1 software and analysed using GeneSpring software v12.1. Statistical analysis was carried out using an unpaired t-test and a fold change with cut-off value >2 with a P-value ≤0.05 was considered to indicate a statistically significant difference.

### Quantitative reverse transcription PCR (qRT-PCR)

qRT-PCR was carried otu to validate the results from microarray analysis and performed in two steps. The high capacity RNA-to-cDNA kit protocol (Applied Biosystems, Foster City, CA, USA) was followed to transcribe 2 μg total RNA into single-stranded cDNA. This was followed by amplification using a thermal cycler (Eppendrof, Hamburg, Germany). The ΔC_T_ of the thymoquinone-treated cells was subtracted from the ΔC_T_ of the untreated cells to determine the differences (ΔΔC_T_) and fold change (2^−ΔΔCT^). The human glyceraldehyde-3-phosphate dehydrogenase (*GAPDH*) reference gene was used as an endogenous control to normalize the fluorescence signals in the untreated and treated cells. Five dilution points were prepared in triplicate for each primer to plot the relative standard curve.

Amplification plots and the standard curve of each target gene and endogenous control were created to determine the primer efficiency. Three biological replicates of thymoquinone-treated samples and three biological replicates of untreated samples were examined. The StepOnePlus Real-Time PCR instrument (Applied Biosystems) was used to run this experiment with a 96-well system. The 5′ nuclease assay (TaqMan probe; Life Technologies Corp., Carlsbad, CA, USA) was used to detect the specific PCR product. It was labelled with the reporter fluorescence dye 6-carboxyfluorescein (6-FAM) at the 5′ end and the quencher dye MGBNFQ at the 3′ end. The fast mode was selected to run the experiment for 40 min. The temperature was adjusted to the one recommended in the manufacturer’s instructions (Applied Biosystems,).

The comparative C_T_ (ΔΔC_T_) method was selected to determine the amount of target nucleic acid sequence in each sample relative to the untreated samples. The normalization of the ΔC_T_ value of the thymoquinone-treated cells to the ΔC_T_ value of the untreated cells was carried out to determine the ΔΔC_T_ value.

## Results

### Quantification and data filtration

Filtering was carried out to minimise errors and generated 24,971 entities. There were seven entities out of 577 entities with a fold change of >2.0 and and P-value ≤0.05.

### Hierarchial clustering

**Figure 1:**
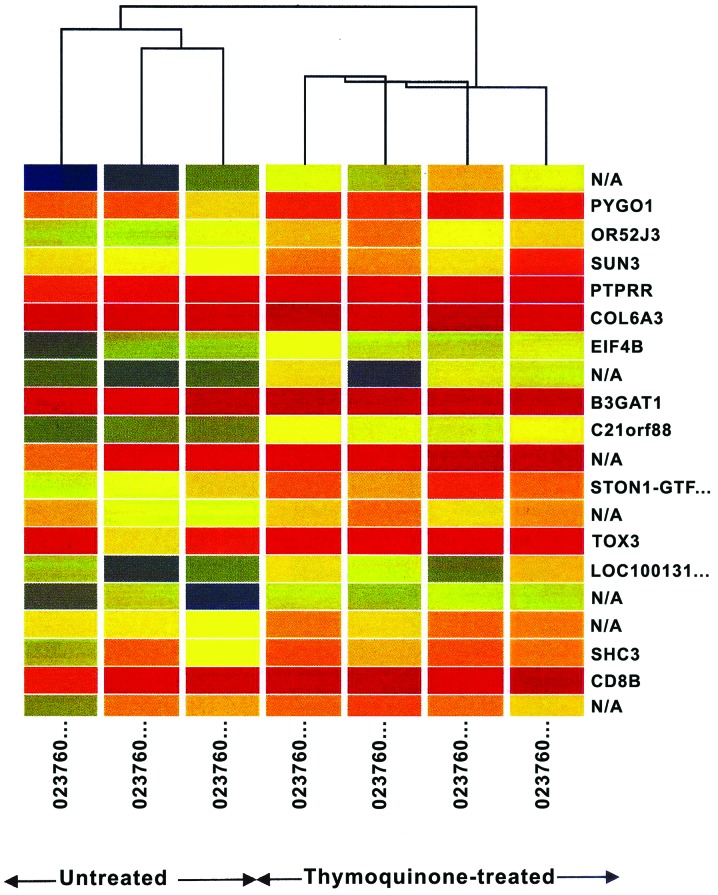
A partial view of hierarchial clustering under both treatment conditions (untreated and thymoquinone-treated cells).

Clustering was carried out to identify entities that were grouped within a cluster which are co-regulated and functionally related. Clustering was carried out on both entities and conditions. This created a two-dimensional dendogram. The untreated samples had a similar expression profile, which linked to form a group. A similar pattern was observed in the thymoquinone-treated samples (Fig. 1).

### Gene Ontology (GO) analysis

**Figure 2:**
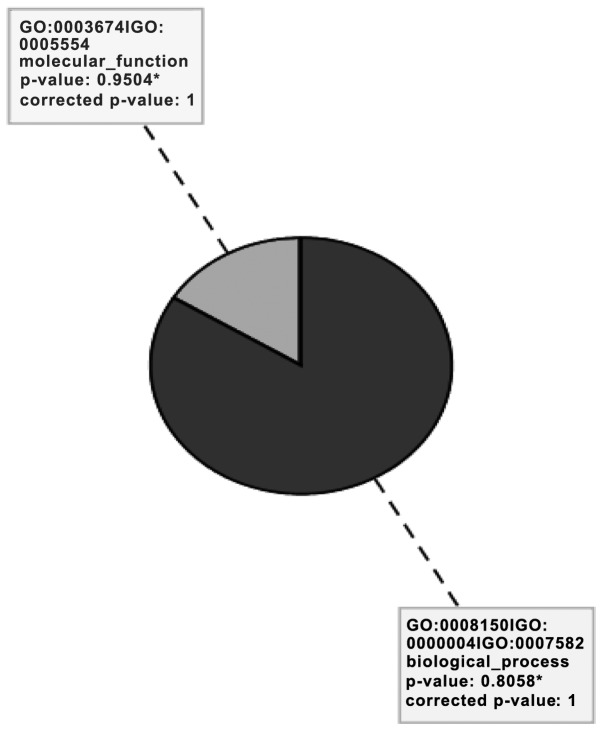
Gene Ontology showing the percentage of genes involved in biological processes and molecular functions.

GO describes gene products in terms of their associated cellular component and molecular function and biological process (Fig. 2). An analysis revealed eight significant GO terms related to biological processes (84%) and molecular functions (16%), as presented in Table I.

### Single experiment analysis (SEA)

SEA identifies matching pathways within the experiment and identifies pathways related to regulated genes. An analysis of the 577 entities with a P≤0.05 and a fold change of >2.0 showed 504 pathways. There were three matched entities in cytochrome p450 (CYP450) (63 entities), phase I and II drug metabolism (49 entities) and interferon type I (54 entities).

Table II presents the downregulated pathways with the highest number of matched entities (16 entities). These were the metapathway biotransformation and interferon signaling pathway (six entities), although these were not significant (P>0.05). The most significant results were observed for the phase I metabolic pathway (P<0.04). The upregulated pathways with the highest number of matched entities were also the metapathway biotransformation (four entities). The most significant results were observed for the GPCR class B secretion pathway (P=0.009).

The most significantly upregulated genes were large intergenic non-coding RNAs (lincRNAs) and the probe was annotated with its genomic location (Table III). The protein tyrosine phosphatase, receptor type, R (*PTPRR*) gene was upregulated by 2.2-fold. SEA revealed genes in the estrogen pathway that were downregulated following treatment with thymoquinone (Table IV). The genes that were downregulated were UDP glucuronosyltransferase 1 family, polypeptide A8 (*UGT1A8*) (by −16.26-fold), cytochrome P450, family 1, subfamily A, polypeptide 1 (*CYP1A1*) (by −2.22-fold), cytochrome P450, family 1, subfamily B, polypeptide 1 (*CYP1B1*) (by −2.23-fold) and NAD(P)H dehydrogenase, quinone 1 (*NQO1*) (by −2.07-fold). Genes in the interferon α and β signaling pathways were also downregulated following treatment with thymoquinone; these genes were the interferon-induced protein with tetratricopeptide repeats (*IFIT*)*1* (by −10.65-fold), *IFIT2* (by −4.20-fold), *IFIT3* (by −5.27-fold), interferon, α-inducible protein (*IFI)6 (*also known as *G1P3*) (-by 7.86-fold) and *IFI27* (by −3.19-fold). The genes downregulated in the type II interferon signaling pathway, included interferon regulatory factor 9 (*IRF9*) (−2.01), *IFIT2* (−4.20), 2′-5′-oligoadenylate synthetase 1, 40/46 kDa (*OAS1)* (−3.5) and *IFI6* (−7.86).

### Expansion and pathway enrichment analysis

**Figure 3:**
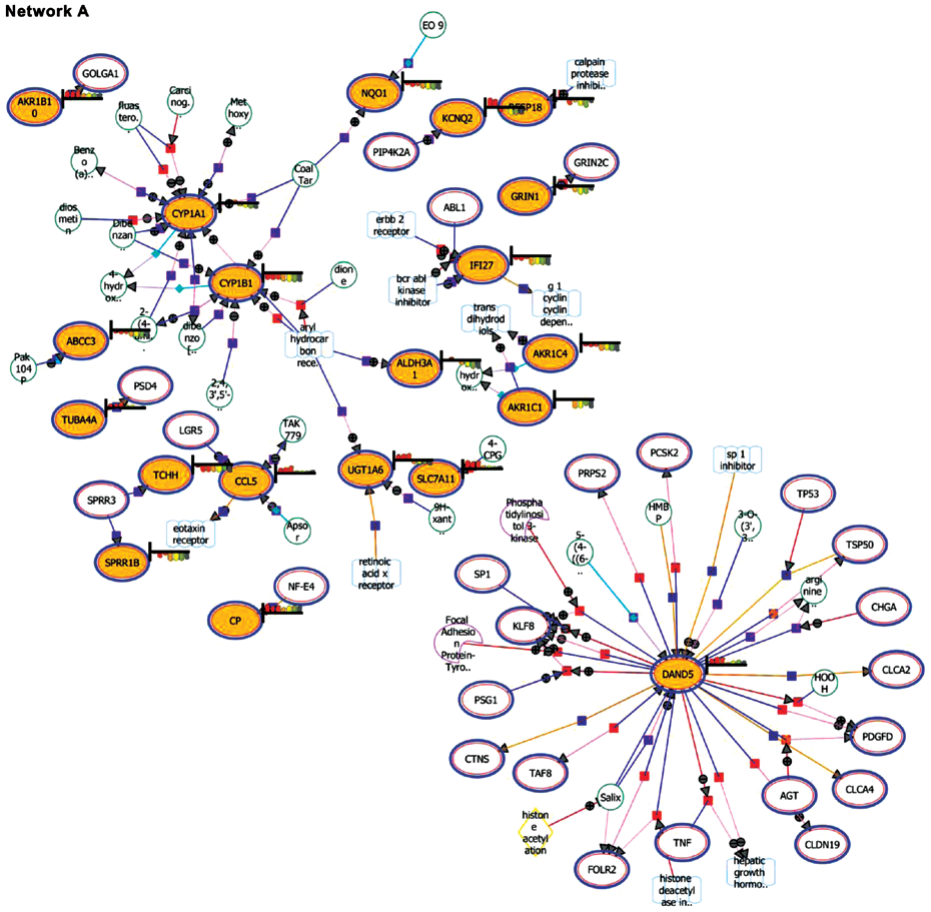

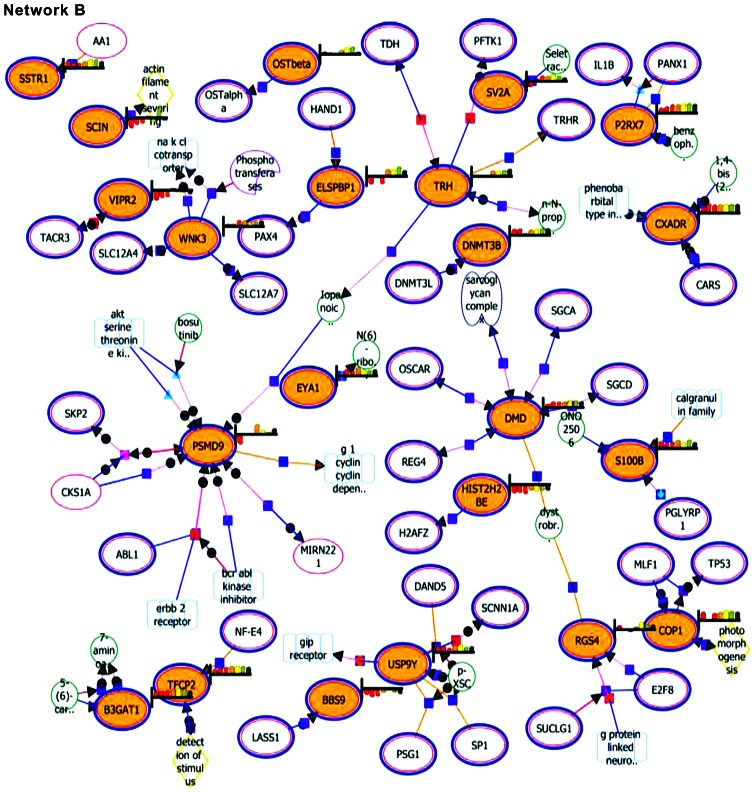
Network A, interactions of downregulated genes. Network B, interactions of upregulated genes.

Expansion and pathway enrichment analysis provided information on the biological functions of the entities. Network A (downregulated genes) showed the DAN domain family member 5, BMP antagonist (*DAND5*) as the focal point among the matched entities (Fig. 3). In addition, *CYP1A1* and *CYP1B1* were also noted to be highlighted in the interaction. Network B (upregulated genes) highlighted the involvement of regulator of G-protein signaling 4 (*RGS4*), proteasome (prosome, macropain) 26S subunit, non-ATPase, 9 (*PSMD9*), ubiquitin specific peptidase 9, Y-linked (*USP9Y*) and dystrophin (*DMD*).

### Validation of thymoquinone regulation of gene expression by qRT-PCR

The efficiency of each primer was determined using the relative standard curve method. The optimal concentration based on the efficiency of the primers was selected for relative quantification (RQ). The ΔΔC_T_ value of *CYP1A1*, solute carrier family 7 (anionic amino acid transporter light chain, xc-system), member 11 (*SLC7A11*) and *UGT1A8* showed a downregulation of 43-, 15-, and 11-fold, respectively. However, the ΔΔC_T_ value of the protein tyrosine phosphatase, receptor type, R (*PTPRR*) and caveolin 1, caveolae protein, 22 kDa (*CAV1*) genes showed an upregulation of 2.2- and 1.5-fold, respectively.

## Discussion

Almost 75% of breast cancer patients are estrogen receptor-positive (6). Tamoxifen is a selective estrogen-receptor modulator. It has been shown to reduce invasive breast cancer risk by 50% (7,8). Despite advances in treatment, there is a need to investigate the use of new drugs to modulate the expression of the estrogen receptor in estrogen receptor-positive breast cancer cells. Furthermore, recurrence develops after certain durations of treatment. In this study, cDNA microarray technology was used to measure the expression levels of all genes within the human genome. Microarray data analysis provided information on the genes and pathways targeted by 50 μM thymoquinone following 24 h of treatment.

Steroid hormones play a main role in the maintenance of normal female function and are normally produced by ovaries (9). Estradiol has been known to be the main and most active endogenous estrogen in pre-menopausal women. It is present in the form of estrone in post-menopausal women (10) and in pregnant women it is in the form of estriol (11). It has been reported that elevated circulating estrogen levels cause increased breast cancer risk (12).

CYP450 enzymes are haem-containing enzymes which catalyse phase 1 drug metabolism (13). *CYP1A1* is expressed in extrahepatic tissues, such as mammary glands, the prostate, ovaries, uterus, colon, testis, adrenal, thymus and lungs (14). The overexpression of *CYP1B1* has been reported in mammary tumours and breast cancer (15–17). The enzymes are present in the estrogen metabolic pathway and are known as 17β-estradiol (E2) hydroxylases (18). The conversion of estradiol to 2-hydroxyestradiol (2-OHE_2_) and 4-hydroxyestradiol (4-OHE_2_) is influenced by the *CYP1A1* and *CYP1B1* genes, respectively (19,20). 4-OHE_2_ is known as the most carcinogenic metabolite of estrogen (21,22). It has been reported to be a vital factor in hormonal carcinogenesis (23). The *CYPIAI* gene regulates the metabolism of estradiol into 2-hydroxyestradiol. It is also involved in the metabolism of estrone to 4-hydroxyestrone (4-OHE_1_). 4-OHE_1_ is known as one of the most potent carcinogenic estrogen metabolites (20). Our findings revealed a downregulation in the expression of the *CYP1A1* and *CYP1B1* genes. Further validation with qRT-PCR revealed a significant downregulation of the *CYPIAI* gene by 43-fold. The downregulation of CYP450 enzymes has been reported to suppress estradiol-2,3-quinone (E_2_-2,3-Q) and estradiol-3,4-quinone (E_2_-3,4-Q) activity (22,24).

Previous studies have reported the anticancer and anti-angiogenic effects of 2- and 4-methoxyestradiol (2- and 4-MeOE_2_) (20,25). Increased levels of the methoxy form of estrone have been shown to have anticancer and anti-angiogenic potential in humans (20). The formation of 2- and 4-MeOE_2_ occurs through the methylation of 2-OHE_2_ and 4-OHE_2_. The oral administration of 2-MeOE_2_ in phase I and phase II clinical trials has been shown to be well tolerated by patients. Its anti-proliferative effects have been demonstrated in nasopharngeal carcinoma and tumor-derived uterine leiomyoma cell lines (26,27). The phase I enzyme UGT1A8 plays a key role in drug metabolism. In this study, we observed a significant downregulation of the *UGT1A8* gene by 16.26-fold. The validation using real-time PCR on relative gene expression revealed a downregulation of 11-fold. The downregulation of this gene would retain 2-MeOE_2_ levels.

Multidrug resistance is the most important mechanism by which cancer chemotherapeutic drugs fail to take effect and resistance develops against anticancer drugs (28,29). The IFN-related DNA damage resistance signature (IRDS) is associated with resistance to chemotherapy or radiation in various cancer cell lines. Some of the IRDS genes are *STAT1*, *ISG15* and *IFIT1*. They are associated with the interferon signaling pathway (30). The upregulation of these genes has been reported in different types of cancer. In our study, whole genome microarray data analysis revealed that the *IFIT1* gene was downregulated by 10.65-fold. In addition, the *IFIT2* and *IFIT3* genes were downregulated by 5.32- and 5.27-fold, respectively. The overexpression of the *IFIT3* and *IFI27* genes has been reported to induced tumour proliferation, angiogenesis and chemoresistance in pancreatic carcinoma cells (32). The findings of our study revealed the downregulation of the *IFI27* and *IFI6* genes by 3.19- and 7.86-fold, respectively following treatment with thymoquinone. The downregulation of the *IFI6* or *GIP3* gene has been shown to reduce MCF-7 cell growth (33).

Interferon-stimulated gene factor 3 (*ISGF3*) is regulated by interferon α. *ISGF3* genes consist of *STAT1*, *STAT2* and *IRF9*. It has been grouped as *ISGF3-α* and *ISGF3-γ*(34,35). *ISGF3-γ* functions through type I interferons (IFN-α and IFN-β) (31). The overexpression of *IRF9* has been observed in almost half of breast and uterine tumours. It may be connected to downstream mediators of interferon signaling to drug resistance (31). In this study, thymoquinone induced the downregulation of *ISGF3* (*IRF9*) by 2.01-fold. A previous study using cDNA microarray analysis demonstrated a high expression of the *STAT1* and *STAT2* genes in MCF-7 cells that overexpressed *IRF9*(31). The downregulation of *IRF9* gene expression may induce the lower expression of interferon-inducible genes (31). This finding further supports the chemopreventive effects of thymoquinone. *STAT1* is commonly overexpressed in breast cancer. It is found to be related with increased resistance to radiation and chemotherapy (36). In breast cancer, the increase in estrogen levels leads to a higher expression of *STAT1* gene (36).

Glutathione (GSH) is known as a compound that promotes drug-resistance through the removal of free radicals (37). The *SLC7A11* gene functions in chemoresistance through the maintenance of intracellular GSH levels (28). In this study, we observed a downregulation of *SLC7A11* gene expression by 12.99-fold. Validation using real-time PCR of relative gene expression revealed a downregulation of 15-fold.

The *PTPRR* gene is known as an inhibitor of MAPK (38). We found that *PTPRR* was upregulated by 2.20-fold and the myocyte enhancer factor 2C (*MEF2C*) gene by 2.22-fold, which promotes apoptosis. A previous study demonstrated that the *PTPRR* gene exerts an inhibitory effect on p44/42 MAPK signaling and transcription factor AP_1_ expression in cervical cancer (39). In this study, we found that the *caspase-10* gene, which is known as one of the initiator caspases, was upregulated by 1.57-fold. Previously, caspase cascade activation has been shown to involve caspase-3, -7 and -9, and not caspase-8 in MCF7/DOX cells (40).

In conclusion, our findings suggest that thymoquinone acts in a synergistic manner involving the estrogen metabolic and interferon pathways as indicated by our microarray findings. The co-adminstration of thymoquinone and tamoxifen requires further investigation in order to fully elucidate the treatment outcomes.

## Figures and Tables

**Table I:** Gene Ontology categories of biological processes and molecular functions.

Gene Ontology categories	Total genes	P-value
Biological processes		
Type I interferon-mediated signaling pathway	62	0.002
Cellular response to type I interferon	62	0.002
Response to type I interferon	63	0.002
Xenobiotic metabolic process	129	0.021
Cellular response to xenobiotic stimulus	130	0.021
Response to xenobiotic stimulus	131	0.021
Molecular functions		
Androsterone dehydrogenase activity	3	0.023
Aldo-keto reductase (NADP) activity	18	0.045

**Table II:** Regulated pathways identified by single experiment analysis.

Pathways	P-value	Entities	Matched entities
Downregulated pathways			
HS_Metapathway_biotransformation	2.38	188	16
HS_Benzo(a)pyrene_metabolism	1.25	9	5
HS_Interferon_α_β_signaling	2.00	26	6
HS_AhR_pathway	4.32	28	6
HS_Estrogen_metabolism	2.48	18	4
HS_Oxidative stress	7.25	30	4
HS_Keap1_Nrf2_pathway	8.92	14	3
HS_Tamoxifen_metabolism	2.17	21	3
HS_Glucuronidation	0.007	26	3
HS_Type_II_interferon_signaling (IFNG)	1.68	37	4
HS_Iron_metabolism_in_placenta	0.003	12	2
HS_Tryptophan_metabolism	0.026	47	3
HS_Phase_I_-_functionalization_of_compounds	0.004	49	3
HS_Glutathione_metabolism	0.008	20	2
HS_Spingolipid_metabolism	0.034	30	2
HS_Interferon_type_I_	0.040	54	3
HS_Hypothetical_Network_for_Drug_Addiction	0.023	32	2
HS_Bile_acid_and_bile_salt_metabolism	0.041	24	2
HS_Nicotine_Activity_on_Chromaffin_Cells	0.029	4	1
HS_Cytochrome_P450	0.009	63	3
Upregulated pathways			
Hs_One_Carbon_Metabolism	0.013	27	2
HS_GPCRs, _Class_B_secretin_like	0.009	23	2
HS_miRs_in_Muscles_Cell_Differentiation	0.015	40	2
HS_Metapathway_biotransformation	0.024	188	4
HS_Thyroxine_thyroid_hormone_production	0.032	5	1
HS_Heart_Development	0.033	47	2
HS_Cell_surface_interaction_at_vascular_wall	0.020	39	2

**Table III:** List of genes which were upregulated following with treatment thymoquinone.

Gene symbol	Description	Fold change
*XLOC001537*	BROAD lincRNAs version v2	6.31
*CARD16*	*Homo sapiens* caspase recruitment domain family, member 16	5.25
*C2CD4B*	*Homo sapiens* C2 calcium-dependent domain containing 4B	4.31
*UBQLNL*	*Homo sapiens* ubiquilin-like	4.21
*MTR*	Methyltetrahydrofolate-homocysteine methyltransferase	3.91
*TMEM100*	Protein 100, transcript variant 2	3.70
*MOBKL2B*	Binder kinase activator-like 2B	3.67
*RNF17*	(RNF17), transcript variant 1	3.62
*STON1GTF2*	*Homo sapiens* ring finger protein 17, transcript variant 1	3.60
*OR52J3*	*Homo sapiens* olfactory receptor, family 52, subfamily J, member 3	3.42
*LOC100133130*	*Homo sapiens* clone FLB4246 PRO1102 mRNA, complete cds	3.30
*NCRNA00261*	*Homo sapiens* non-protein coding RNA 261, non-coding	3.28
*FBXW2*	*Homo sapiens* F-box and WD repeat domain containing 2	3.26
*LOC100131763*	*Homo sapiens* cDNA FL137602 fis, clone BRCOC2009380	3.19
*SCIN*	*Homo sapiens* scinderin, transcript variant 2	3.19
*PGAP1*	*Homo sapiens* post-GPI attachment to protein 1	3.17
*S100B*	*Homo sapiens* S100 calcium binding protein B (S100B)	3.16
*SV2A*	*Homo sapiens* synaptic vesicle glycoprotein 2A	3.16
*C7orf54*	*Homo sapiens* chromosome 7 open reading frame 54, non-coding RNA	3.15
*FAM65B*	*Homo sapiens* family with sequence similarity 65, member B, transcript variant 1	3.15

**Table IV:** List of genes which were downregulated following treatment with thymoquinone.

Gene symbol	Description	Fold change
*UGT1A8*	*Homo sapiens* UDP glucuronosyltransferase 1 family, polypeptide A8	−16.26
*SLC7A11*	*Homo sapiens* solute carrier family 7 (anionic amino acid transporter light chain, xc-system), member 11	−12.99
*IFIT1*	*Homo sapiens* interferon-induced protein with tetatricopeptide repeats 1, transcript variant 2	−10.65
*IF16*	*Homo sapiens* interfero, α-inducible protein 6, transcript variant 3	−7.86
*C17orf64*	*Homo sapiens* chromosome 17 open reading frame 64	−6.20
*ALDH3A1*	*Homo sapiens* aldehyde dehydrogenase 3 family, member A1, transcript variant 2	−5.85
*UGT1A6*	*Homo sapiens* UDP glucuronosyltransferase 1 family, polypeptide A6, transcript variant 1	−5.68
*IFIT2*	*Homo sapiens* interferon-induced protein with tetratricopeptide repeats 2	−5.32
*IFIT3*	*Homo sapiens* interferon-induced protein with tetratricopeptide repeats 3, transcript variant 1	−5.27
*HMOX1*	*Homo sapiens* hemeoxygenase (decycling) 1	−5.13
*DNAH6*	*Homo sapiens* dynein, axonemal, heavy chain 6	−4.58
*TPRG1*	*Homo sapiens* tumour protein p63 regulated 1	−4.55
*PCLO*	*Homo sapiens* piccolo (presynaptic cytomatrix protein), transcript variant 1	−4.33
*CHD5*	*Homo sapiens* chromodomain helicase DNA binding protein 5	−4.28
*CCR1*	*Homo sapiens* chemokine (C-C motif) receptor 1	−4.26
*AKR1C1*	*Homo sapiens* aldo-ketoreductase family 1, member C1 (dihydrodiol dehydrogenase 1; 20-α (3-α)-hydroxysteroid dehydrogenase)	−4.21
*GSTA5*	*Homo sapiens* glutathione S-transferase α 5	−4.21
*AKR1B15*	*Homo sapiens* aldo-ketoreductase family 1, member B15	−4.13
*HEATR7B1*	HEAT repeat containing 7B1	−3.98
*GPX2*	*Homo sapiens* glutathione peroxidase 2 (gastrointestinal)	−3.88
